# IDENTIFYING AND PREDICTING PLACE OF CARE TRAJECTORIES DURING THE LAST THREE YEARS OF LIFE

**DOI:** 10.1093/geroni/igad104.1466

**Published:** 2023-12-21

**Authors:** Haiqun Lin, Anum Zafar, Soko Setoguchi, Olga Jarrín

**Affiliations:** Rutgers, The State University of New Jersey, Newark, New Jersey, United States; Rutgers, The State University of New Jersey, New Brunswick, New Jersey, United States; Rutgers, The State University of New Jersey, New Brunswick, New Jersey, United States; Rutgers, The State University of New Jersey, New Brunswick, New Jersey, United States

## Abstract

Aging in place is a goal for most older adults, however in presence of advanced illness and/or Alzheimer’s disease and related dementia (ADRD) institutional placement may be needed. The number of days in each care setting in each quarter of the last three years of life was determined through linkage of several Medicare datasets. Using group-based modeling we examined place of care trajectories during the last three years of life among a 10% sample of Medicare beneficiaries who died in 2018 (n=199,828), providing a pre-COVID19 baseline for future studies, to identify dual trajectories of inpatient/institutional care, and skilled home healthcare/home hospice. Nine distinct trajectory classes and their associated sociodemographic and clinical characteristics were identified. The first three classes were characterized by healthcare use concentrated in the last three months of life (comprising 29.1%, 20.5%, and 9.1% of the sample); the next three classes were characterized by low use of inpatient/institutional care with long-term use of skilled home healthcare/hospice (14.2%, 8.2%, and 4.8%); and the remaining inpatient/institutional classes characterized by low use of skilled home healthcare/hospice with long-term use of inpatient/institutional care (4.8%, 4.9%, and 4.6%). The beneficiaries in the remaining sample are assigned to a place of care trajectory class using the parameter estimates derived from the 10% sample without further model fitting. We investigate the prediction utility of risk factors in multiple domains of demographic, clinical, and social determinants of health. Our findings may help to inform care preference during last period of life understanding the associated factors.

